# Susceptibility of Canada Geese (*Branta canadensis*) to Highly Pathogenic Avian Influenza Virus (H5N1)

**DOI:** 10.3201/eid1312.070502

**Published:** 2007-12

**Authors:** John Pasick, Yohannes Berhane, Carissa Embury-Hyatt, John Copps, Helen Kehler, Katherine Handel, Shawn Babiuk, Kathleen Hooper-McGrevy, Yan Li, Quynh Mai Le, Song Lien Phuong

**Affiliations:** * Canadian Food Inspection Agency, Winnipeg, Manitoba, Canada; † Public Health Agency of Canada, Winnipeg, Manitoba, Canada; ‡ National Institute of Hygiene and Epidemiology, Hanoi, Vietnam; § National Center for Veterinary Diagnosis, Hanoi, Vietnam

**Keywords:** Influenza A virus, (H5N1) subtype, pathogenesis, immunity, research

## Abstract

Migratory birds have been implicated in the long-range spread of highly pathogenic avian influenza (HPAI) A virus (H5N1) from Asia to Europe and Africa. Although sampling of healthy wild birds representing a large number of species has not identified possible carriers of influenza virus (H5N1) into Europe, surveillance of dead and sick birds has demonstrated mute (*Cygnus olor*) and whooper (*C. cygnus)* swans as potential sentinels. Because of concerns that migratory birds could spread H5N1 subtype to the Western Hemisphere and lead to its establishment within free-living avian populations, experimental studies have addressed the susceptibility of several indigenous North American duck and gull species. We examined the susceptibility of Canada geese (*Branta canadensis*) to HPAI virus (H5N1). Large populations of this species can be found in periagricultural and periurban settings and thus may be of potential epidemiologic importance if H5N1 subtype were to establish itself in North American wild bird populations**.**

## Abstract 2

Prior exposure of Canada geese to a North American low pathogenic virus (H5N2) decreases their susceptibility to Eurasian highly pathogenic avian influenza virus (H5N1).

Wild aquatic birds belonging to the orders Anseriformes and Charadriiformes have long been recognized as the natural reservoirs for all influenza type A viruses (*1*). Spread from such wild birds to domestic poultry and various mammalian species occurs intermittently. Most viruses that initially infect domestic poultry will replicate only within respiratory or digestive tracts and cause no or very mild disease, referred to as low-pathogenic avian influenza (LPAI) (*2*). However, once introduced into domestic poultry, some viruses of the H5 and H7 hemagglutinin (HA) subtypes can mutate to a highly pathogenic form, producing a systemic infection referred to as highly pathogenic avian influenza (HPAI) (*2*). The hypothesis that HPAI H5 and H7 viruses emerge from low-pathogenic precursors only after the H5 and H7 LPAI precursors have been introduced into domestic poultry has been supported by work demonstrating that HPAI viruses do not appear to form separate phylogenetic lineages in waterfowl (*3*). Except for A/tern/South Africa/1961 (H5N3), no evidence existed before 2002 that an HPAI virus could cause deaths or be maintained within wild bird populations.

In late 2003, an HPAI (H5N1) outbreak of unprecedented magnitude began in Southeast Asia. Approximately 1 year before this, a high mortality rate attributed to HPAI virus (H5N1) was observed in waterfowl and other wild birds in Hong Kong (*4*). This led to speculation that wild birds may have contributed to the virus spread. In the spring of 2005, mass dieoffs of wild birds occurred at Qinghai Lake, People’s Republic of China (*5*,*6*), an event heralded as the beginning of the long-range spread of HPAI (H5N1) from Asia into Europe and subsequently Africa, with migratory birds implicated as playing a role (*7*,*8*). Identifying which species of birds were involved in this spread is not only of academic interest but also of practical importance to surveillance activities because of concerns that migratory birds could also introduce H5N1 subtype into the Western Hemisphere. We examined the susceptibility of Canada geese (*Branta canadensis)* to infection with an HPAI virus (H5N1) and the effect that pre-exposure to an LPAI virus (H5N2) has on clinical disease, pathology, and virus shedding.

## Materials and Methods

### Viruses

The influenza viruses used in this study included A/chicken/Vietnam/14/2005 (H5N1) and A/mallard/British Columbia/373/2005 (H5N2). Vietnam/05 stocks were grown and titrated on Japanese quail fibrosarcoma (QT-35) cells. This isolate bears a PQRERRRKR/GLF HA_0_ cleavage site (GenBank accession no. EF535027), has an intravenous pathogenicity index of 2.97, and produced a 100% mortality rate in oronasally inoculated leghorn chickens receiving 10^5^, 10^4^, and 10^3^ PFU by 3, 4, and 6 days postinfection (dpi), respectively. British Columbia/05 stocks were grown and titrated in 9-day-old chicken embryos. Prior characterization of this isolate demonstrated that it has a PQRETR/GLF HA_0_ cleavage site (GenBank accession no. DQ826532) typical for LPAI viruses.

### Animals

Twenty-two Canada geese were captured with the permission of Environment Canada (Canadian Wildlife Service permit no. CWS06-M009) and were handled and cared for in accordance with Canadian Council on Animal Care guidelines and the animal use protocol approved by the Institutional Animal Care Committee. The geese consisted of 11 adult (6 male + 5 female) and 11 young-of-year (6 male + 5 female) birds. The latter were estimated to be ≈40 days of age at capture. Adult and juvenile birds were randomly assembled into 3 experimental groups, and each group subsequently housed in separate Biosafety Level-3 biocontainment cubicles: 1) a control group comprising 1 juvenile + 1 adult bird, 2) a pre-exposure group comprising 5 juvenile + 5 adult birds, and 3) a naive group comprising 5 juvenile + 5 adult birds.

After a 3-week acclimation period, the pre-exposure group was inoculated with 10^6^ 50% egg infectious dose (EID_50_) of British Columbia/05 applied to the nares, oral cavity, and cloaca. Twenty-eight days later, pre-exposure and naïve groups were challenged with 1.7 × 10^5^ PFU of Vietnam/05 applied to the nares, oral cavity, and eye. The control group received a sham inoculum of minimal essential medium. Timed necropsies involving 1 juvenile and 1 adult bird from pre-exposure and naïve groups were performed on days 3 and 6 postchallenge (dpc). All remaining birds were either humanely euthanized when moribund or allowed to survive until 20 or 21 days if they showed mild disease or remained clinically normal.

### ELISA and Hemagglutination-Inhibition (HI) Assays

Group A specific nucleoprotein (NP) antibodies were detected with a competitive ELISA as described previously (*9*). H5-specific antibodies were detected by microtiter plate HI test that used 4 HA U of A/duck/British Columbia/26–6/2005 (H5N2) and chicken erythrocytes.

### Virus Neutralization Assay

We incubated 200 EID_50_ of Vietnam/05 with an equal volume of 2-fold serially diluted test serum (1:4 to 1:512), incubated for 60 min at 37°C, and then used it to inoculate 9-day-old chicken embryos through the allantoic cavity. Egg deaths and HA titers were monitored and virus neutralization titers determined.

### Real-Time Reverse Transcription–PCR (RT-PCR) Assays

Specimens were stored at –70°C before RNA was extracted. Total RNA was extracted from 0.5 mL of 10% (wt/vol) tissue emulsions or clarified swab specimens by using an RNeasy Mini Kit (QIAGEN, Mississauga, Ontario, Canada). A semiquantitative real-time RT-PCR (*10*) that targets the M1 gene of influenza A virus segment 7 was conducted. Full-length, in vitro transcribed segment 7 RNA, serially diluted in buffer, was run with each assay to give a semiquantitative estimate of the viral load in each tissue.

### Immunohistochemistry

Formalin-fixed, deparaffinized, and rehydrated 5-μm tissue sections were quenched for 10 min in aqueous 3% H_2_O_2_, rinsed in MilliQ water, and placed into Tris-buffered saline plus Tween (TBST) buffer for 5 min. Sections were pretreated with proteolytic enzyme (DakoCytomation, Carpinteria, CA, USA) for 15 min, rinsed twice with TBST, and incubated for 1 h with a monoclonal antibody specific for influenza A nucleoprotein (Clone 1331, Biodesign, Sasco, ME, USA) at a dilution of 1:5,000. The sections were washed with TBST, then incubated for 30 min with the Envision + anti-mouse (horse radish peroxidase–labeled) polymer kit (DakoCytomation), followed by a TBST rinse. Diaminobenzidine was used as the substrate chromagen, and slides were counterstained with Gill’s hematoxylin.

## Results

### A/mallard/British Columbia/373/2005 (H5N2) Pre-Exposure

Upon arrival, 12 of 12 juvenile geese tested negative and 10 of 12 adult geese tested positive for influenza A virus NP antibodies (Table 1). To determine the HA subtype specificity of the seropositive birds, HI assays were run with 4 HA U of the following antigens: H1N1 (A/Ck/BC/3/98); H2N9 (A/Pintail/AB/293/77); H4N6 (A/Dk/BC/14/99); H5N2 (A/mallard/BC/373/05); H6N1 (A/Tk/ON/844–2/04); and H7N3 (A/Ck/BC/514/04). All tests were negative, indicating that the birds did not appear to have pre-existing H5-specific antibodies. Real-time RT-PCR–negative cloacal swab specimens indicated that the birds were also not actively infected.

**Table 1:** NP and H5 antibody levels in juvenile and adult Canada geese*

Animal ID	0 dpi cELISA (NP % inhibition)	0 dpi H5 HI assay†	14 dpi (H5N2) cELISA (NP % inhibition)	21 dpi (H5N2) H5 HI assay†	20–21 dpi (H5N1) cELISA (NP)	20–21 dpi (H5N1) H5 HI assay†
Juveniles						
852S/27R	Neg (20)	<8	Pos (66)	16	Euthanized or died‡	
853S/28R	Neg (13)	<8	Pos (64)	64	Pos (46% inhibition)	8
856S/31R	Neg (21)	<8	Pos (57)	256	Euthanized	
858S/33R	Neg (22)	<8	Pos (55)	128	Pos (48% inhibition)	64
859S/34R	Neg (19)	<8	Pos (49)	256	Euthanized	
851S/26R	Neg (24)	<8			Euthanized	
854S/29R	Neg (23)	<8			Euthanized	
855S/30R	Neg (22)	<8			Euthanized	
860S/35R	Neg (24)	<8			Euthanized	
861S/36R	Neg (20)	<8				
857S/32Y	Neg (18)	<8				
Adults						
842S/42Y	Pos (93)	<8	Pos (99)	512	Euthanized	
844S/44Y	Neg (23)	<8	Pos (99)	64	Pos (46% inhibition)	<8
845S/45Y	Pos (58)	<8	Pos (96)	8	Euthanized	
846S/46Y	Pos (76)	<8	Pos (96)	<8	Pos (63% inhibition)	<8
847S/47Y	Pos (74)	<8	Pos (99)	ND	Euthanized	
840S/40Y	Pos (45)	<8			Pos (99% inhibition)	>4,096
841S/41Y	Neg (22)	<8			Euthanized	
843S/43Y	Pos (78)	<8			Euthanized	
848S/48Y	Pos (39)	<8			Pos (98% inhibition)	64
849S/49Y	Pos (93)	<8			Pos (98% inhibition)	32
850S/50Y	Pos (85)	<8				

*NP, nucleoprotein; cELISA, competitive ELISA; dpi, days postinfection; Neg, negative (<30% inhibition); Pos, positive (≥30% inhibition); HI, hemagglutinin inhibition; ND, not determined. **†**4 hemagglutinin units of A/duck/British Columbia/26–6/2005 (H5N2) used in assay. ‡Euthanized or died before day 20–21 postinoculation with virus (H5N1).

After inoculation with 10^6^ EID_50_ of British Columbia/05, all birds remained clinically normal. The juvenile birds gained weight, but 3 of 5 adult birds had a 6%–10% loss of bodyweight after infection. Cloacal swabs from juvenile birds were real-time RT-PCR positive at 3 dpi; swabs from adult birds were negative (oropharynegeal swabs not tested). At 6 and 10 dpi, cloacal and oropharyngeal swabs from both juvenile and adult birds were real-time RT-PCR negative, indicating that viral shedding was brief. Although most of the British Columbia/05 infected birds developed H5-specific HI antibody titers (Table 1), these sera did not neutralize Vietnam/05 in a chicken embryo–based neutralization assay.

### A/chicken/Vietnam/14/2005 (H5N1) Challenge

Twenty-eight days after pre-exposure to British Columbia/05, birds in the pre-exposure and naïve groups were challenged with Vietnam/05. Juvenile birds were estimated to be 13 weeks of age at this time. Adult birds in the British Columbia/05 pre-exposure group exhibited mild decreases in feed consumption and mild depression 5–7 dpc. Except for 1 bird with a positive oropharyngeal swab sample at 6 dpc, oropharyngeal and cloacal swab specimens for the adults tested real-time RT-PCR negative at 2, 3, and 6 dpc. Juvenile birds in the British Columbia/05 pre-exposure group exhibited clinical signs similar to those of the adults with the addition of transient nervous signs manifested as repetitive jerking head movements. Viral shedding, as determined by real-time RT-PCR and confirmed by isolation, was detected at 3 dpc in oropharyngeal swab samples in 3 of 5 birds and in a cloacal swab sample in 1 of 5 birds. Complete necropsies showed no gross lesions in juvenile or adult birds at 3, 6, 11, and 21 dpc. The cerebrum, brain stem, and spinal cord of juvenile birds exhibited low levels of viral nucleic acid at 11 and 21 dpc (Appendix Table). Other organs were weakly positive by real-time RT-PCR to varying degrees.

In contrast, juvenile birds in the naïve group showed 100% morbidity after Vietnam/05 challenge; clinical signs included severe depression, inappetence, bright yellow diarrhea, ruffled feathers, hunched posture, repetitive jerking head movements, weakness, staggering gait, distressed vocalization, wing droop, and terminal coma. All birds died or were humanely euthanized by 5 dpc. Viral nucleic acid was detected in the oropharyngeal swab specimens collected at all time points before euthanasia or death; cloacal swab specimens were not as consistently positive. Adult birds also showed 100% morbidity but with clinical signs and viral shedding less pronounced than that observed in juveniles. Necropsies were performed on 2 adults on days 3 and 5; the remaining 3 birds survived until 20 dpc.

Gross pathologic lesions included congestion of the mucosal surface of the trachea, edema and multifocal pinpoint hemorrhages on the serosal surface of the pancreas, splenomegaly, hemorrhage within the ceca, conjunctivitis, congestion of the meninges and cerebral blood vessels, and hemorrhages on the surface of the brain. Virtually all tissues collected from juvenile birds in the naïve group were real-time RT-PCR positive; heaviest viral loads were found in cerebrum, brain stem, and spinal cord. Adult bird 841S/41Y, which required euthanasia at 5 dpi, also had levels of viral nucleic acid in the central nervous system (CNS) comparable to those found in naïve juveniles. This was one of the adult birds with no pre-existing NP antibodies at the beginning of the acclimation period (Table 1). Viral nucleic acid was found in the CNS of a second adult (840S/40Y), euthanized at 20 dpc, but at levels that were 5–7 logs lower than those found in juveniles or the adult bird euthanized at 5 dpc.

**Figure 1:**
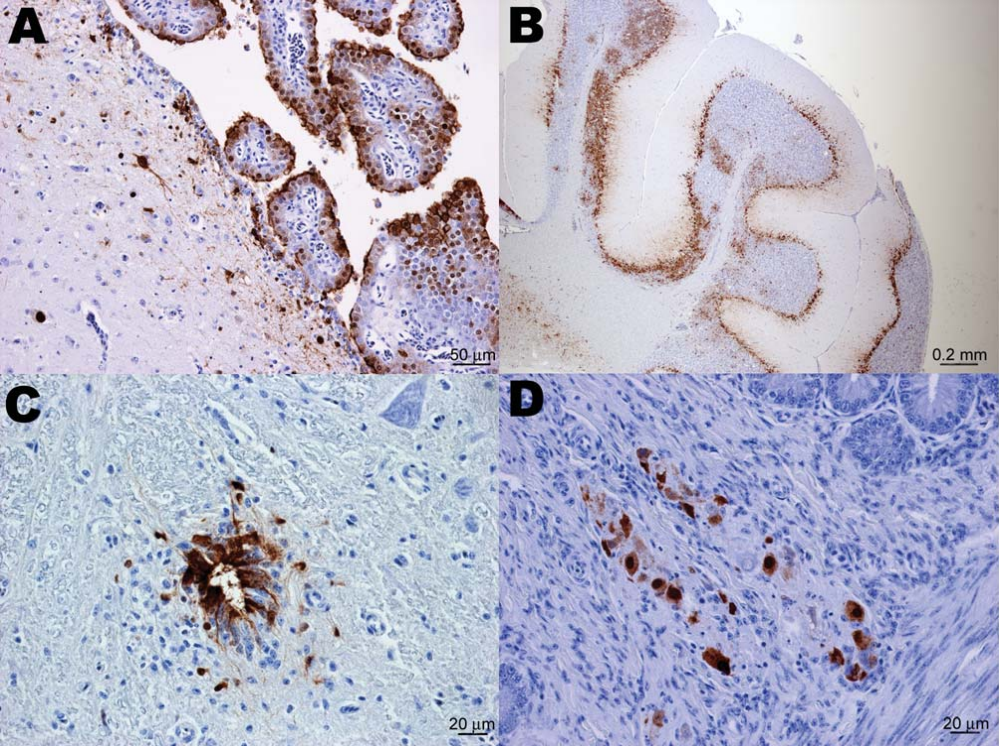
Immunohistochemical staining for influenza virus nucleoprotein in central and peripheral nervous system of naive juvenile Canada geese tissues after challenge with influenza virus (H5N1). A) Cerebrum. Positive immunolabeling of neurons, glial cells, ependymal and choroid plexus epithelial cells. B) Cerebellum. Extensive positive immunolabeling of Purkinje cells and neurons of the granular layer. C) Spinal cord. Positive immunolabeling of ependymal cells of the central canal and adjacent neurons and glial cells. D) Small intestine. Positive immunolabeling of neurons of the submucosal plexus.

**Figure 2:**
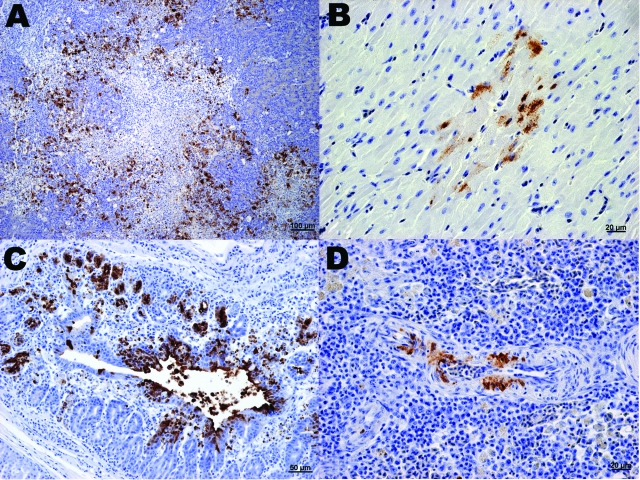
Immunohistochemical (IHC) staining for influenza virus nucleoprotein in tissues of naïve juvenile Canada geese after challenge with influenza virus (H5N1). A) Pancreas. Large areas of necrosis are surrounded by pancreatic acinar cells with strong positive intranuclear and intracytoplasmic immunolabeling. B) Heart. Positive intranuclear and intracytoplasmic immunolabeling of myocytes. C) Proventriculus. Strong positive immunolabeling of compound tubular gland epithelium. D) Splenic arteriole. Positive IHC staining of vascular smooth muscle cells.

Specific influenza A virus immunolabeling was found in all tissues collected from naïve juvenile birds (Table 2). The most consistently affected tissues were the brain, spinal cord, parasympathetic ganglia of the gastrointestinal tract, heart, and pancreas (Figures 1, 2). Within the small intestine and cecum, the strongest and most consistent immunolabeling involved the parasympathetic ganglia of the submucosal and myenteric plexi (Figure 1, panel D) with only the occasional scattered smooth muscle and vascular endothelial cell within the gut mucosa positive for viral antigen. In the 3 birds in which the proventriculus was affected, viral antigen was detected in numerous cell types, including both surface columnar and glandular epithelium, smooth muscle cells of the muscularis mucosa, vascular smooth muscle, and the parasympathetic ganglia (Figure 2, panel C). In the lungs, antigen could be identified in a few capillary endothelial cells. Positive immunolabeling within trachea, liver, kidney, and breast muscle was minimal and observed in only a few birds. Immunohistochemical analysis of tissues collected from naive adult birds detected specific immunolabeling in only 1 bird (841S/41Y) euthanized at 5 dpc; tissues and cells affected were similar to those observed in naive juveniles.

**Table 2:** Distribution of influenza virus antigen in tissues of naïve juvenile Canada geese tissues after challenge with influenza virus (H5N1)

Tissue	Animal 861S/36R dpi 3*	Animal 855S/30R dpi 4*	Animal 851S/26R dpi 5*	Animal 854S/29R dpi 5*	Animal 860S/35R dpi 5*	IHC-positive cell types
Trachea	+	+	–	–	–	Vascular endothelium
Lung	++	++	–	+	–	Vascular endothelium, mononuclear cells
Esophagus	++	++	+	+	–	Epithelium, Vascular smooth muscle, Smooth muscle of muscularis externa, Mucous glands
Proventriculus	+	+	–	++	–	Epithelium (columnar, glandular), muscularis mucosa, vascular smooth muscle, parasympathetic ganglia
Ventriculus	++	++	–	++	–	Epithelium
Gut	++	+++	–	++	+	Parasympathetic ganglia, mucosal smooth muscle, vascular endothelium
Cecal tonsil	++	++	+	++	–	Parasympathetic ganglia
Pancreas	++	+++	++	++	+	Exocrine acinar cells
Liver	–	–	–	+	–	Hepatocytes
Spleen	++	–	+	–	–	Vascular smooth muscle, mononuclear cells
Kidney	+	–	–	–	–	Tubular epithelium
Muscle	+	+	–	–	–	Vascular smooth muscle
Heart	++	++	++	+	+	Myocytes
Brain	++	+++	+++	+++	+++	Neurons, glial cells, ependymal cells, choroid plexus epithelium
Spinal cord	+	++	++	++	++	Ependymal cells, neurons, glial cells, leptomeninges
Sciatic nerve	+	–	–	–	–	Vascular endothelium
Brachial nerve	–	–	–	–	–	None

*Numbers of immunohistochemically positive cells: +, few; ++, moderate; +++, numerous; –, virus antigen negative; dpi, days postinfection.

## Discussion

Deaths of mute *(Cygnus* *olor*) and whooper (*C.* *cygnus*) swans have signaled the arrival of HPAI virus (H5N1) in Europe (*11*,*12*). The affected swans had nervous signs that included somnolence, incoordination, and ataxia (*11*) and gross pathology that included multifocal hemorrhagic necrosis in the pancreas, pulmonary congestion and edema, and subepicardial hemorrhages (*13*). Recent studies addressing the susceptibility of North American waterfowl species to HPAI virus (H5N1) have shown wood ducks (*Aix sponsa*) and laughing gulls (*Larus atriculla*) to be highly susceptible, while mallards (*Anas platyrhnchos*), northern pintails (*A. acuta*), blue-wing teals (*A. crecca*) and redheads (*Aythya Americana*) to be refractory (*14*,*15*). Previous reports from Asia (*4*) and Europe (*13*) have indicated that HPAI virus (H5N1) can produce deaths in naturally infected Canada geese. Our study supports these observations and further demonstrates this susceptibility to be dependent on the age and immunologic status of the animal.

Adult birds were generally more resistant to Vietnam/05 than juveniles, regardless of which experimental group they belonged to. Although results of this study indicate that prior infection with a North American LPAI virus (H5N2) protects juvenile Canada geese against a lethal H5N1 subtype challenge, the mechanism responsible is unresolved. Although HI titers in poultry strongly correlate with protection against virulent challenge from viruses expressing the same HA subtype (*16*), the ability of British Columbia/05 H5-specific antibodies to neutralize Vietnam/05 in vitro was not demonstrated. British Columbia/05 and Vietnam/05 have 84% amino acid similarity in their HA_1_ subunits. The receptor binding domain (*17*), which comprises an α-helix (190-helix, HA_1_ 188–190) and 2 loop structures (130-loop, HA_1_ 134 to 138, and 220-loop, HA_1_ 221 to 228) in addition to residues Tyr^96^, Trp^153^, and His^183^ is remarkably conserved for both viruses. Multiple amino acid differences that cluster around the receptor-binding domain (data not shown) may explain the inability of British Columbia/05 antisera to neutralize Vietnam/05 in vitro. Recent reports (*18*,*19*) have suggested that prior infection with viruses expressing heterologous HA subtypes can also protect chickens against a lethal (H5N1) challenge. Protection against HPAI virus (H5N1) in chickens that were previously infected with an H9N2 subtype correlated with the proportion of pulmonary CD8^+^ T cells expressing gamma interferon (*19*). The hypothesis that cell-mediated immunity may have played a role in affording protection to the birds in this study is supported by the observation that even though NP antibody–positive naive adults did not appear to possess H5-specific antibodies, they were resistant to Vietnam/05 challenge.

The pronounced neurotropism that Vietnam/05 exhibited for Canada geese is similar to that reported for other susceptible wild bird species (*13*–*15*). A unique finding in our study was the widespread involvement of gastrointestinal parasympathetic ganglia. This has not been previously reported for wild birds, to our knowledge, although viral antigen within the parasympathetic ganglia of the small intestine of experimentally infected ducks has been documented (*14*). The mechanism by which avian influenza viruses invade the CNS has been most thoroughly investigated with mouse models (*20*–*22*). These studies have shown that after intranasal inoculation, neurotropic influenza A viruses can invade the CNS of mice by spreading along peripheral nerves; viral antigen is mainly detected in the vagal and trigeminal nuclei of the brainstem but not in the cerebral cortex. A compartmentalized mouse dorsal root ganglion neuron culture system (*22*) has further demonstrated that influenza A viruses could infect the distal parts of axons and reach the neuronal cell bodies by retrograde axonal transport in a microtubule-independent fashion. The involvement of the parasympathetic ganglia in our geese suggests that CNS infection may occur by transmission of influenza virus via autonomic nerves to their centers in the brain stem. In contrast to the situation in mice, there is a more diffuse infection of cortical and midbrain neurons as well as choroid and ependymal epithelial cells. The latter may indicate that a hematogenous route involving penetration of the blood–brain barrier with infection propagated to glial cells and neurons (*23*) may also be involved.

Our work has demonstrated that Canada geese, and in particular immunologically naïve, young-of-year animals, may be suitable targets for dead bird surveillance activities. Based on our experiments, HPAI virus (H5N1) can be expected to produce pronounced neurologic signs and high deaths in this age group. CNS, pancreas, and heart specimens can be used in PCR or immunohistochemical diagnosis. However, prior exposure to North American lineage H5 viruses specifically, or avian influenza viruses of other HA subtypes more generally, may protect juvenile and adult geese against a virulent H5N1 subtype challenge, hence complicating detection. Determining the mechanism responsible for this apparent cross-protection will require further research.

## Supplementary Material

Appendix Table Real-time RT-PCR analysis of Canada geese tissues following challenge with influenza virus (H5N1)*
