# Supplementary material for: Susceptibility of Canada Geese (Branta canadensis) to Highly Pathogenic Avian Influenza Virus (H5N1)
Source: Emerg Infect Dis. 2007 Dec;13(12):1821–7. doi: 10.3201/eid1312.070502 (PMC2876756; doi:10.3201/eid1312.070502)
Supplement: Appendix Table — Real-time RT-PCR analysis of Canada geese tissues following challenge with influenza virus (H5N1)* [file 07-0502_appT-s1.pdf]

Appendix Table. Real-time RT-PCR analysis of Canada geese tissues following challenge with influenza virus (H5N1)*

Group	Animal	dpi	Trachea	Lung	Proventriculus	Gut	Cecal tonsils	Pancreas	Liver	Spleen	Kidney	Muscle	Heart	Cerebrum	Brainstem	Spinalcord
H5N2 juveniles																
	859S/34R	3	Neg	Neg	Neg	Neg	Neg	Neg	Neg	Neg	Neg	Neg	Neg	Neg	Neg	Neg
	852S/27R	6	Neg	3.6	Neg	Neg	Neg	Neg	Neg	Neg	Neg	Neg	Neg	Neg	Neg	Neg
	856S/31R	11	Neg	Neg	Neg	Neg	Neg	Neg	Neg	Neg	Neg	Neg	Neg	Neg	3.4	3.3
	853S/28R	21	Neg	3.8	2.9	2.4	3.1	Neg	Neg	2.9	Neg	Neg	Neg	3.8	4.2	3.0
	858S/33R	21	Neg	Neg	3.1	Neg	Neg	Neg	2.9	Neg	2.5	Neg	2.8	2.9	Neg	2.9
H5N2 adults																
	842S/42Y	3	Neg	Neg	Neg	Neg	Neg	Neg	Neg	Neg	Neg	Neg	Neg	Neg	3.3	Neg
	845S/45Y	6	Neg	Neg	Neg	Neg	Neg	Neg	Neg	Neg	Neg	Neg	Neg	Neg	Neg	Neg
	847S/47Y	11	Neg	Neg	Neg	Neg	Neg	Neg	Neg	Neg	Neg	Neg	Neg	Neg	Neg	3.5
	844S/44Y	21	2.7	2.5	Neg	Neg	Neg	2.2	2.9	3.0	2.7	Neg	Neg	Neg	Neg	Neg
	846S/46Y	21	2.8	2.9	Neg	Neg	Neg	Neg	Neg	2.8	Neg	Neg	2.4	3.1	2.9	3.0
Naïve juveniles																
	861S/36R	3	7.4	4.0	5.5	5.8	6.0	4.6	Neg	5.2	5.7	4.9	Neg	9.0	8.5	7.1
	855S/30R	4	4.2	4.5	Neg	3.2	3.1	4.6	3.3	Neg	3.5	4.0	3.6	7.6	5.8	6.4
	851S/26R	5	6.2	3.9	5.7	4.3	6.0	4.5	3.8	4.0	5.9	Neg	3.8	10.3	9.1	7.7
	854S/29R	5	6.4	5.9	6.3	4.9	5.8	5.4	3.1	3.9	Neg	Neg	4.9	10.4	7.7	7.9
	860S/35R	5	4.6	4.4	4.4	5.7	4.4	4.5	2.9	2.5	2.6	3.2	Neg	9.5	8.6	7.3
Naïve adults																
	843S/43Y	3	Neg	Neg	Neg	Neg	Neg	Neg	Neg	Neg	Neg	Neg	Neg	Neg	3.1	Neg
	841S/41Y†	5	4.6	5.8	6.2	5.7	5.6	6.5	3.8	Neg	5.8	4.7	6.4	9.5	8.3	7.8
	840S/40Y	20	Neg	Neg	Neg	2.7	Neg	2.8	2.2	2.3	Neg	Neg	Neg	2.9	2.8	3.1
	848S/48Y	20	Neg	Neg	Neg	Neg	Neg	Neg	Neg	Neg	Neg	Neg	Neg	Neg	Neg	Neg
	849S/49Y	20	Neg	Neg	Neg	Neg	Neg	Neg	Neg	Neg	Neg	Neg	Neg	Neg	Neg	Neg
Controls																
	850S/50Y	13	Neg	Neg	Neg	Neg	Neg	Neg	Neg	Neg	Neg	Neg	Neg	Neg	Neg	Neg
	857S/32Y	13	Neg	Neg	Neg	Neg	Neg	Neg	Neg	Neg	Neg	Neg	Neg	Neg	Neg	Neg

*RT PR, reverse transcription–PCR. All real-time RT-PCR results expressed as $\log_{10}$ viral RNA copy number per gram of tissue.

†Animal tested negative for nucleoprotein antibodies at beginning of acclimation period.
